# Relationship between Toxoplasma Gondii and Psychotic Disorders with Implications toward a Brain-based Diagnostic System and Novel Treatment Approaches: A Study Protocol

**DOI:** 10.9734/JAMMR/2021/v33i1330958

**Published:** 2021-06-08

**Authors:** Dina Raja, Shyamanta Das, Chimanjita Phukan, Simanta Talukdar, Samrat Singh Bhandari, Naba Kumar Hazarika, Dipesh Bhagabati

**Affiliations:** 1 Department of Microbiology, Gauhati Medical College Hospital, Guwahati, Assam, India.; 2 Department of Psychiatry, Gauhati Medical College Hospital, Guwahati, Assam, India.; 3 Department of Microbiology, Assam Medical College Hospital, Dibrugarh, Assam, India.; 4 Department of Psychiatry, Assam Medical College Hospital, Dibrugarh, Assam, India.; 5 Department of Psychiatry, Sikkim Manipal Institute of Medical Sciences, Gangtok, Sikkim, India.; 6 Hindlabs centres, HLL Lifecare Ltd, Assam CM’s Free Lab Diagnostic Service Project, India.; 7 Department of Psychiatry, Agartala Govt. Medical College & G.B. Pant Hospital, Agartala, Tripura, India.

**Keywords:** Toxoplasma gondii, psychotic disorders, diagnostic system, seropositivity rate

## Abstract

What role infectious agents play in the causation of psychotic disorders? To investigate this area, we have aimed to investigate the relationship between Toxoplasma gondii and psychotic disorders. A hospital-based cross-sectional study is designed. IgM and IgG antibodies to T. gondii in patients with psychotic disorders will be measured and presented in result. Seropositivity rates will be compared with first-degree relatives and healthy volunteers. Also, types of psychotic disorders and seropositivity rate will be compared. Here, we are presenting the study protocol with implications toward a brain-based diagnostic system and novel treatment approaches.

## 1. INTRODUCTION

### 1.1 Background and Rationale

The role that infectious agents play in the aetiology of psychotic disorders is an area of interest. Among such candidates, Toxoplasma gondii becomes a prominent one. Evidences are emerging for this line of thinking [1]. Antibodies to T. gondii are more in patients with psychotic disorders. Some adults with toxoplasmosis exhibit psychotic symptoms. Epidemiological similarities are observed in toxoplasmosis and psychotic disorders. Antipsychotic agents inhibit T. gondii [2]. Toxoplasma raises dopamine levels in animals. Childhood exposure to cats is high in patients with psychotic disorders.

Abdollahian et al. [3] found more prevalence of T. gondii infection among schizophrenia patients compared to control in Iran. Chen et al. [4] found increased seropositivity of anti-Toxoplasma immunoglobulin G (IgG) and immunoglobulin M (IgM) not only with schizophrenia but also with bipolar disorder in China. By demonstrating elevated Toxoplasma exposure in recent onset psychosis, Yolken et al. [5] gave new insight to temporal relationship between exposure and disease onset.

The relationship of T. gondii and psychotic disorders has the potential to establish a brain-based diagnostic system in psychiatry and pave the way for novel therapeutic options with disease modifying effect.

## 2. OBJECTIVES

### 2.1 Specific Objectives

1. Measurement of IgM and IgG antibodies to T. gondii in patients with psychotic disorders.
2. Comparison of the seropositivity rate for anti-Toxoplasma IgG and IgM antibodies among patients with psychotic disorders with that of first-degree relatives (FDR) as well as healthy volunteers (HV).
3. Comparison of the types of psychotic disorders and seropositivity rate.

### 2.2 Hypotheses

A group of patients with psychotic disorders have serological evidence of Toxoplasma infection. There are certain characteristic clinical and demographic variables among patients with psychotic disorders who have serological evidence of Toxoplasma infection.

There might be a relationship between toxoplasmosis and the aetiology of schizophrenia, and an understanding of the pathogenesis of Toxoplasma infections in individuals with schizophrenia might lead to new approaches to the management of this disorder.

### 2.3 Design

The study will be a hospital-based cross-sectional study.

## 3. MATERIALS AND METHOD

### 3.1 Participants, Interventions, and Outcomes

#### 3.1.1 Study setting

Department of Psychiatry and Department of Microbiology, Gauhati Medical College Hospital (GMCH), Guwahati, Assam, India.

### 3.2 Eligibility Criteria

#### 3.2.1 Inclusion criteria for participants

Patients with psychosis as a defining feature (schizophrenia, acute and transient psychotic disorder, and delusional disorder) and psychosis as an associated symptom (mood disorder and substance use disorder).

#### 3.2.2 Exclusion criteria for participants

Delirium, dementia, mental retardation, and neurological disorders that would affect cognitive performance including epilepsy, a history of encephalitis or head trauma, or any other reported disorder of the central nervous system.

#### 3.2.3 Eligibility criteria for study centres

Patients with psychotic disorders attend for diagnosis and treatment, e.g. in Department of Psychiatry, GMCH.

IgG and IgM antibodies to T. gondii are measured, e.g. in Department of Microbiology, GMCH.

#### 3.2.4 Eligibility criteria for who will perform

Competency in diagnosis and treatment of psychotic disorders, e.g. second, fourth, fifth, and seventh authors are psychiatrists.

Competency in measurement of IgG and IgM antibodies to T. gondii, e.g. first, third, and fifth authors are microbiologists.

#### 3.2.5 Outcomes

Demography of participants. Clinical information of patients. IgM and IgG antibodies to T. gondii.

#### 3.2.6 Participant timeline

One-year.

### 3.3 Sample Size

The sample size is calculated taking the prevalence to be estimated at 60% that gives the maximum sample size, with 95% levels of confidence and 20% bound on error of estimation. Taking this prevalence and stated permissible level of error, the sample size for the study is calculated using the formula

n=4×pq/d2

where n = required sample size, p = 0.6, q = 0.4, d = 20%.

Considering a 20% attrition rate, the sample size is 65 + 13 = 78.

### 3.4 Recruitment

Laboratory technician attending the individuals for collection of blood sample, without the individuals having to go to the laboratory is a strategy to improve adherence and procedure for monitoring adherence.

### 3.5 Data Collection, Management, and Analysis

#### 3.5.1 Data collection methods

After written consent by the participant and her family, demographic and clinical information as well as 5 ml of blood samples will be taken from patients with psychotic disorders, first-degree relatives (FDR), and healthy volunteers (HV). The samples will be centrifuged for 5 minutes at 3,500 rpm; separated, quantitated, and stored at −20°C until later analysis. The serum obtained will be screened for IgM and IgG anti-Toxoplasma antibodies using the Enzyme Linked Fluorescent Assay (ELFA) in the mini VIDAS system (BioMérieux).The samples will be processed according to manufacturer’s instruction. The diagnoses of the patients with psychotic disorders will be made according to the criteria of the tenth revision of the World Health Organizatioin’s International Statistical Classification of Diseases and Related Health Problems (ICD-10) [6].

Relevant care and interventions for which the patients with psychotic disorders have attended Department of Psychiatry, GMCH are permitted to continue concomitantly. Participant/guardian request and worsening disease leading to clinically uncooperative state are criteria for discontinuing.

#### 3.5.2 Statistical methods

Data will be analysed by descriptive (frequency and percentage) and inferential (Fisher’s exact and chi-square tests) statistics using Statistical Package for the Social Sciences (SPSS) and InStat GraphPad.

## 4. RESULT

**Fig. 1.**
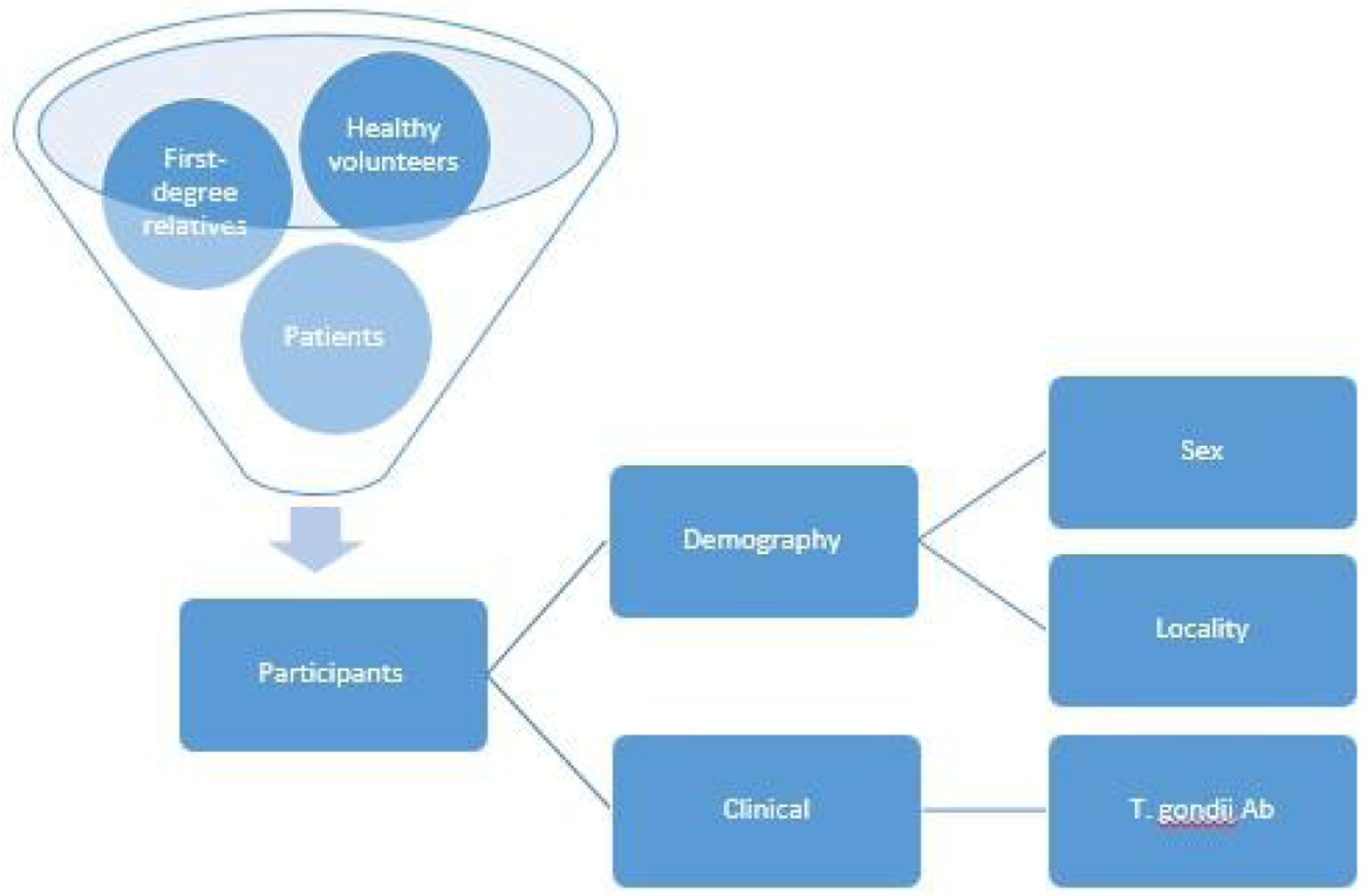
Schematic diagram. T. gondii Ab: Antibodies to Toxoplasma gondii

Fig. 1. schematically represents the study procedure. Participants would constitute of patients, FDR, and HV. Their demography and clinical data would be collected. Sex and locality would be the demography while clinical data would be the antibodies to T. gondii.

Table 1 will show characteristics of the sample. Characteristics would include sex, locality, diagnosis, participant distribution, IgM and IgG status.

Tables 2 and 3 will show seroprevalence of anti-T. gondii antibodies in different participants. The Ig status can be positive, negative, or equivocal.

Tables 4 and 5 will show seroprevalence of anti-T. gondii antibodies in different groups. Diagnosis would be according to ICD-10:[6] F1 standing for mental and behavioural disorders due to psychoactive substance use, F2 standing for schizophrenia, schizotypal and delusional disorder, and F3 standing for mood [affective] disorders.

## 5. DISCUSSION

We have presented here the study protocol of the investigation of the relationship between Toxoplasma gondii and psychotic disorders with implications toward a brain-based diagnostic system and novel treatment approaches.

This line of investigation is not restricted to psychosis among adults. Researchers have explored this subject in different neuropsychiatric disorders as well as in the young population. Abd El-Aal et al. [7] found “significant associations between T. gondii seropositivity of epilepsy and depression groups where youth and adults had the highest sero-T. gondii infection especially male in rural areas with low social class”. Among children and adolescents, Yalın Sapmaz et al. [8] found higher T.gondii IgG seropositivity in depression, especially with suicidality. But, exploring suicidality among adolescents and T. gondii infection, Sari and Kara [9] found no significant differences between the patient and the control groups.

## 6. CONCLUSION

This study protocol outlines how the investigation of the relationship between Toxoplasma gondii and psychotic disorders is planned that has implications toward a brain-based diagnostic system and novel treatment approaches.

## Figures and Tables

**Table 1.** Characteristics of the sample

Characteristics	N	%
**Sex**		
Women	x	x
Men	x	x
**Locality**		
Rural	x	x
Urban	x	x
**Diagnosis**		
F1	x	x
F2	x	x
F3	x	x
**Participant distribution**		
Patient	x	x
First-degree relative	x	x
Healthy volunteer	x	x
**IgM status**		
Negative	x	x
Positive	x	x
Equivocal	x	x
**IgG status**		
Negative	x	x
Positive	x	x
Equivocal	x	x

F1: Mental and behavioural disorders due to psychoactive substance use, F2: Schizophrenia, schizotypal and delusional disorders, F3: Mood [affective] disorders

**Table 2.** Seroprevalence of anti-Toxoplasma gondii IgM antibodies in different participants

Participants	IgM status					
	IgM negative		IgM positive		IgM equivocal	
	N	%	N	%	N	%
Patient	x	x	x	x	x	x
FDR	x	x	x	x	x	x
HV	x	x	x	x	x	x
Total	x	x	x	x	x	x

FDR: First-degree relative, HV: Healthy volunteer

**Table 3.** Seroprevalence of anti-Toxoplasma gondii IgG antibodies in different participants

Participants	IgG status					
	IgG negative		IgG positive		IgG equivocal	
	N	%	N	%	N	%
Patient	x	x	x	x	X	x
FDR	x	x	x	x	X	x
HV	x	x	x	x	X	x
Total	x	x	x	x	X	x

FDR: First-degree relative, HV: Healthy volunteer

**Table 4.** Seroprevalence of anti-Toxoplasma gondii IgM antibodies in different groups

Groups	IgM positive		IgM negative		IgM equivocal		Total	
	N	%	N	%	N	%	N	%
F1	x	x	x	x	x	x	x	x
F2	x	x	x	x	x	x	x	x
F3	x	x	x	x	x	x	x	x
None*	x	x	x	x	x	x	x	x
Total	x	x	x	x	x	x	x	x

* The ‘None’ group constitutes of first-degree relatives (FDR) and healthy volunteers (HV). F1: Mental and behavioural disorders due to psychoactive substance use, F2: Schizophrenia, schizotypal and delusional disorders, F3: Mood [affective] disorders

**Table 5.** Seroprevalence of anti-Toxoplasma gondii IgG antibodies in different groups

Groups	IgG positive		IgG negative		IgG equivocal		Total	
	N	%	N	%	N	%	N	%
F1	x	x	x	x	x	x	x	x
F2	x	x	x	x	x	x	x	x
F3	x	x	x	x	x	x	x	x
None*	x	x	x	x	x	x	x	x
Total	x	x	x	x	x	x	x	x

* The ‘None’ group constitutes of first-degree relatives (FDR) and healthy volunteers (HV). F1: Mental and behavioural disorders due to psychoactive substance use, F2: Schizophrenia, schizotypal and delusional disorders, F3: Mood [affective] disorders

## References

[1] Yolken RH, Dickerson FB, Fuller Torrey E. Toxoplasma and schizophrenia. Parasite Immunol. 2009;31(11):706–15. DOI:10.1111/j.1365-3024.2009.01131.x. 19825110

[2] Jones-Brando L, Torrey EF, Yolken R. Drugs used in the treatment of schizophrenia and bipolar disorder inhibit the replication of Toxoplasma gondii. Schizophr Res. 2003;62(3):237–44. DOI: 10.1016/s0920-9964(02)00357-2. 12837520

[3] Abdollahian E, Shafiei R, Mokhber N, Kalantar K, Fata A. Seroepidemiological study of Toxoplasma gondii infection among psychiatric patients in Mashhad, Northeast of Iran. Iran J Parasitol. 2017;12(1):117–22. 28761468 PMC5522687

[4] Chen X, Chen B, Hou X, Zheng C, Yang X, Ke J, Association between Toxoplasma gondii infection and psychiatric disorders in Zhejiang, Southeastern China. Acta Trop. 2019;192:82–6. DOI: 10.1016/j.actatropica. 2019.02.001. Epub 2019 Feb 4. 30731066

[5] Yolken R, Torrey EF, Dickerson F. Evidence of increased exposure to Toxoplasma gondii in individuals with recent onset psychosis but not with established schizophrenia. PLoS Negl Trop Dis. 2017;11(11):e0006040. DOI: 10.1371/journal.pntd.0006040. 29108011 PMC5690692

[6] World Health Organization. The ICD-10 classification of mental and behavioural disorders: clinical descriptions and diagnostic guidelines. Geneva: World Health Organization; 1992.

[7] Abd El-Aal NF, Saber M, Fawzy N, Ashour WR. Sero-prevalence of anti-Toxoplasma gondii antibodies among patients with neuropsychiatric disorders: epilepsy and depression. J Egypt Soc Parasitol. 2016;46(3):729–36. 30230768

[8] Yalın Sapmaz Ş, Şen S, Özkan Y, Kandemir H. Relationship between Toxoplasma gondii seropositivity and depression in children and adolescents. Psychiatry Res. 2019;278:263–7. DOI:10.1016/j.psychres.2019.06.031. Epub 2019 Jun 20. 31238296

[9] Sari SA, Kara A. Association of suicide attempt with seroprevalence of Toxoplasma gondii in adolescents. J Nerv Ment Dis. 2019;207(12):1025–30. DOI:10.1097/NMD.0000000000001046. 31688493

[10] Equator Network. SPIRIT 2013 Statement: Defining standard protocol items for clinical trials; 2021. Accessed 29 March 2021. Available: https://www.equator-network.org/reporting-guidelines/spirit-2013-statement-defining-stand

